# Advances in adaptive laboratory evolution applications for *Escherichia coli*

**DOI:** 10.1016/j.synbio.2025.07.011

**Published:** 2025-07-30

**Authors:** Weixiang Peng, Xi Zhang, Qingsheng Qi, Quanfeng Liang

**Affiliations:** State Key Laboratory of Microbial Technology, Taishan college, School of Life Science, Shandong University, Qingdao, 266237, PR China

**Keywords:** *Escherichia coli*, Adaptive evolution, Synthetic biology, High-throughput sequencing, Mutation, Forward genetics

## Abstract

Adaptive Laboratory Evolution (ALE), a well-established framework in microbial evolution research, is widely applied in synthetic biology. By simulating natural selection through controlled serial culturing, ALE promotes the accumulation of beneficial mutations, leading to the emergence of specific adaptive phenotypes and bypassing the complexities inherent in rational genetic engineering. With advancements in next-generation sequencing and molecular biology, the integration of high-throughput omics and molecular tools with ALE has significantly enhanced the mapping of genotype-phenotype relationships and the characterization of mutational landscapes. This has propelled progress in microbial evolution, biochemical theory, and interdisciplinary applications. *Escherichia coli* (*E. coli*), a premier chassis in synthetic biology, benefits from its genetic tractability and metabolic flexibility, making it an ideal model for ALE studies. This review examines recent developments in ALE applications for *E. coli*, exploring its methodological principles, experimental design paradigms, notable case studies, and synergies with emerging technologies, providing valuable theoretical insights and practical guidance for related research.

## Graphical abstract

**Figure:**
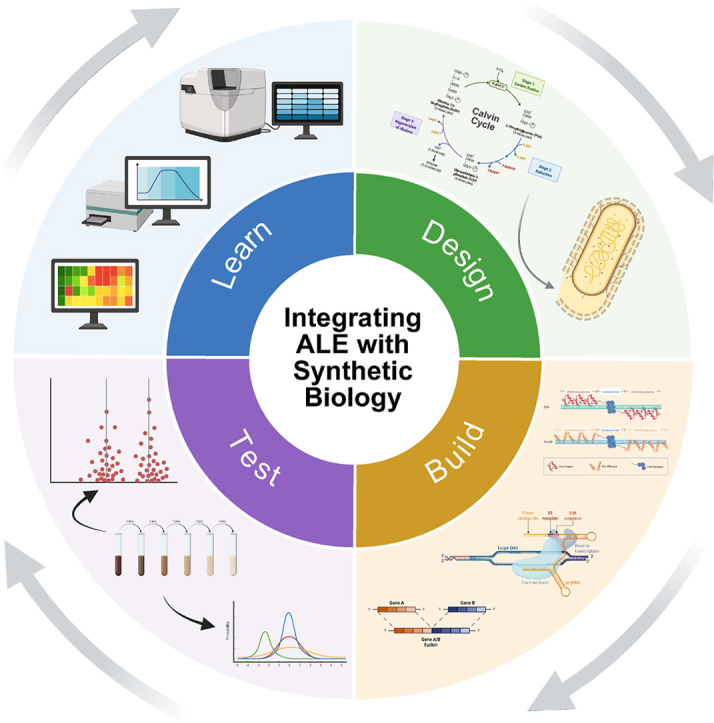

## 1 Introduction

### 1.1 The application needs of adaptive evolution in synthetic biology

Synthetic biology has imposed increasingly complex demands on the targeted modification of microbial chassis cells. *E*. *coli*, with its well-characterized genetic background and rapid division cycle of approximately 20 min, stands as one of the most extensively employed engineered chassis. Despite the availability of established genetic manipulation tools such as CRISPR-Cas9 and MAGE, rational design strategies, including metabolic pathway reconstruction and genome simplification, frequently face unpredictable defects arising from the complexities of the metabolic network. These issues can manifest as energy imbalances, transcription-translation conflicts, or the accumulation of toxic intermediates. Thus, the integration of ALE technology has become increasingly essential, as it leverages natural selection pressures to expedite the phenotypic optimization of strains. This technique offers a distinct solution for validating robustness and achieving functional complementation of chassis cells.

ALE centres on phenotypic optimization through the application of artificial selection pressures, which interact synergistically with the physiological characteristics of *E. coli*. The organism's rapid division time—approximately 20 min per generation—substantially reduces the duration of ALE experiments. For instance, in the evolution of ethanol-tolerant strains, just 80 generations suffice to isolate mutants with a tolerance improvement of at least one order of magnitude [1]. Moreover, the inherent redundancy and plasticity of its metabolic network enable ALE to compensate for the loss of essential pathways through coordinated mutations in multiple genes. For example, in the case of the genome-reduced strain MDS42, ALE enhanced isopropanol tolerance by inducing a mutation in the ppGpp synthetase (*relA*), which mitigated the stringent response under stress conditions [2]. This “irrational design” approach is particularly effective for optimizing complex phenotypes, as it fosters the co-evolution of multiple gene modules without requiring prior identification of genotype-phenotype relationships.

### 1.2 The principles of adaptive evolution and its application advantages

**Fig. 1:**
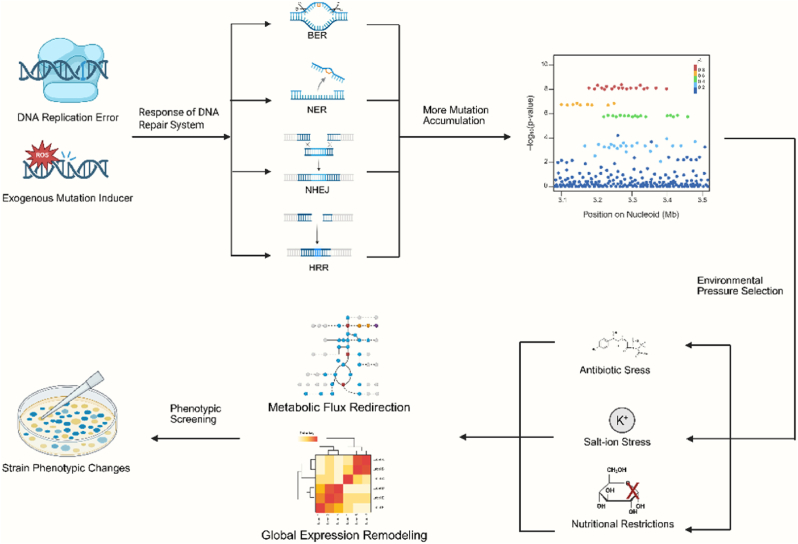
**Core molecular mechanism of ALE.** DNA replication errors and exogenous mutations induce genomic alterations, which are counteracted by repair pathways (e.g., BER, NER, NHEJ, HRR). Under environmental selection pressures, metabolic flux and gene expression are reprogrammed, resulting in the emergence of adaptive phenotype.

The molecular basis of ALE is underpinned by two fundamental mechanisms: the induction of random mutations and the phenotypic screening under selection pressure (Fig. 1). In *E. coli*, mutations primarily arise from DNA replication errors, with a spontaneous mutation rate of approximately 1 × 10^−3^ mutations per gene per generation, as well as from DNA damage repair processes triggered by environmental stresses such as oxidative stress, which activates the SOS response pathway. For instance, the RecBCD complex, essential for repairing double-strand DNA breaks, facilitates the accumulation and selection of mutations through its nuclease and recombination activities during ALE. Upon DNA damage, the SOS response is activated, leading to an upregulation of DNA polymerases IV (Pol IV) and V (Pol V), thus increasing the mutation rate. Through iterative passaging, typically spanning hundreds to thousands of generations, beneficial mutations are selected and accumulated. In long-term glucose limitation experiments, for example, inactivation mutations in *rpoB* and *rpoC* (genes encoding RNA polymerase subunits) are retained, as they enhance growth rates when cultured in glycerol medium [3]. The mutations induced by ALE can be classified into three categories, with their dynamic distribution reflecting the hierarchical regulatory nature of *E. coli* metabolic network: recurrent mutations, reverse mutations, and compensatory mutations [4]. Recurrent mutations refer to the independent acquisition of identical gene mutations in different strains under the same selective pressure, such as the co-occurrence of mutations in *arcA* (encoding an anaerobic respiration regulator) and *cafA* (encoding ribonuclease G) during ethanol tolerance evolution [1]. Reverse mutations optimize phenotypes by restoring ancestral gene functions, as demonstrated by the revertant mutation in the *prfB* gene of the artificially recoded strain C321.ΔA, which restored protein synthesis fidelity [5]. Compensatory mutations, in contrast, facilitate functional substitution through the activation of bypass metabolic pathways, exemplified by the recovery of acetate assimilation in *E. coli* under isobutanol stress [6].

In synthetic biology, ALE is indispensable due to its unparalleled ability to optimize complex phenotypes. For instance, when integrating non-natural metabolic pathways, rational design often fails due to rejection response of the host metabolic network. In contrast, ALE dynamically adjusts selection pressures, identifying mutation combinations that effectively balance heterologous pathway expression with host adaptability. A key example is the work by Gleizer et al. (2019), who constructed an autotrophic *E. coli* strain by activating the Calvin-Benson-Bassham (CBB) cycle *via* ALE. They concurrently optimized the formate dehydrogenase (FDH) to ribulose-1,5-bisphosphate carboxylase (Rubisco) activity ratio, enabling the strain to grow solely on CO_2_ [7]. This process involves multi-level regulation of transmembrane proton gradient maintenance, cofactor regeneration, and carbon flux redistribution, far surpassing the predictive capacity of rational design. Similarly, in antibiotic resistance research, a team from the University of Zurich used CRISPR technology to create a fitness landscape of *E. coli* proteins encompassing 260,000 mutations. They discovered that approximately 75 % of evolutionary pathways could lead to high-resistance phenotypes [8]. This finding challenges the traditional fitness landscape theory, which asserts that “evolutionary pathways are constrained by local peaks”, and highlights *E. coli* capacity for efficient adaptation through synergistic mutations in multiple genes under dynamic selection pressures.

The indispensability of ALE in *E. coli* research is further demonstrated by its role in ‘evolutionary complementation’ to address defects in synthetic biology chassis. Lenski's long-term evolution experiment has shown that *E. coli* can continuously accumulate adaptive mutations under sustained selective pressure. The diversity and unpredictability of its evolutionary trajectories offer a unique perspective for understanding microbial evolutionary dynamics [9]. This feature positions ALE as a powerful tool for deciphering complex environmental adaptation mechanisms, such as tolerance to high osmotic pressure and toxic metabolites. In salidroside synthesis, for instance, a research team successfully screened for tyrosol-tolerant strains through ALE, overcoming growth inhibition caused by intermediate products, thereby facilitating subsequent glycosylation reactions [10]. Furthermore, the combination of ALE with physical mutagenesis techniques, such as heavy ion radiation, has expanded its potential applications. By inducing a synergistic effect between genomic instability and selective pressure, ALE enhances the evolutionary efficiency of target phenotypes [11].

## 2 The experimental approach of ALE in *E. coli*

ALE is a cornerstone technology in directed evolution strategies, where the design of experimental methodologies directly impacts the controllability of the evolutionary trajectory and the efficacy of phenotypic improvements. In *E. coli* ALE research, experimental approaches are typically classified into three main technical modules: continuous transfer culture, automated evolution systems, and retrospective verification. The optimization and integration of these modules provide a standardized framework for understanding the adaptive mechanisms of microorganisms.

### 2.1 Parameter selection and optimization of the continuous transfer model

**Fig. 2:**
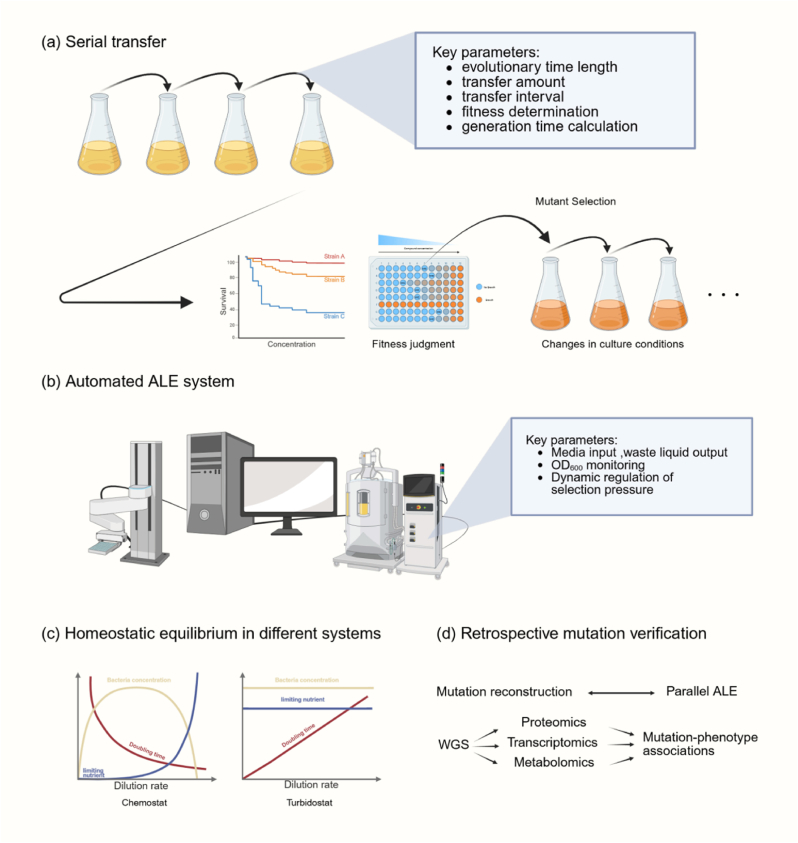
**Experimental model of ALE. (a)** The classic serial transfer method in ALE, involving repeated passaging and fitness evaluation. **(b)** An automated ALE system, offering precise environmental control and real-time monitoring. **(c)** Comparison of homeostatic equilibria in different continuous culture modes (chemostat vs. turbidostat). **(d)** Retrospective mutation verification, combining multi-omics analyses and mutation reconstruction experiments.

Continuous transfer culture forms the basis of the traditional ALE experimental model (Fig. 2a), where the regulation of core parameters directly influences evolutionary dynamics. Key factors include experimental duration, transfer volume, transfer interval, fitness determination, and generation time calculation. The experiment must span a sufficient number of generations to ensure mutation accumulation and phenotypic stability. The Long-Term Evolution Experiment (LTEE) shows that *E. coli* achieves significant phenotypic improvements after 200–400 generations in a carbon-limited medium, although optimization of key metabolic pathways may require extending beyond 1000 generations [12]. Moreover, the evolution of complex phenotypes, such as stress tolerance or substrate co-utilization, often necessitates a staged design approach. For example, a study employed a staged design to progressively increase selection pressure. Initially, sethoxydim was used to inhibit ACCase, promoting lipid synthesis. Subsequently, sesamol was introduced to alleviate lipid synthesis inhibition, enhancing the production of lipids and DHA. This two-step staged design effectively optimized metabolic pathways and promoted the evolution of target phenotypes [13]. The precise regulation of transfer volume has dual effects on maintaining population genetic diversity: a low transfer volume (1 %–5 %) accelerates the fixation of dominant genotypes but risks the loss of low-frequency beneficial mutations, while a high transfer volume (10 %–20 %) preserves greater diversity and supports parallel evolution with multiple objectives. In LTEEs, Lenski et al. observed that a 1 % transfer volume maintains the effective population size within a constant and moderate range, reducing genetic drift and ensuring adequate selection pressure [14]. The transfer time interval must be adjusted dynamically based on the growth rate dynamics of bacterial cells in the culture medium. Typically, transfers occur at the beginning of the stationary phase, with dynamic regulation achieved through OD_600_ measurements or substrate consumption rates. Short intervals, where transfers occur during the logarithmic mid-phase, maintain high growth rate selection pressure, optimizing growth phenotypes. In contrast, longer intervals, transferring during the stationary phase, activate stress response pathways and foster tolerance evolution. In a study on parallel evolution in *E. coli*, it was found that bacterial densities between 5 × 10^6^ and 5 × 10^8^ cells/mL at the time of transfer maximize mutation accumulation efficiency when cells are at the end of the logarithmic growth phase [15]. Adaptability assessment criteria have evolved from a singular growth rate metric to a multidimensional evaluation system. This system integrates specific growth rate (μ), substrate conversion rate (Yx/s), and product synthesis rate (qp) to provide a more comprehensive standard for adaptability indices. Innovations in generation time calculation have improved the accuracy of evolutionary dynamics analysis. Traditionally, ALE experiment duration was determined based on generations; however, a novel approach now utilizes cumulative cell division count (CCD) as the time scale reference [16].

### 2.2 Applications and technological innovations of turbidostat and chemostat

The introduction of the automated ALE system has effectively mitigated the operational variability associated with traditional methods. Notably, the combined use of turbidostat and chemostat systems has become a critical factor in achieving significant technological advancements (Fig. 2b). The chemostat regulates the growth rate by maintaining a constant dilution rate, making it especially useful for studying evolutionary dynamics under specific metabolic flux conditions (Fig. 2c). Its main advantage lies in the ability to analyze the relationship between metabolic flux and adaptive mutations under steady-state culture conditions. For example, Jeong et al. (2016) employed the chemostat in adaptive evolution studies of *E. coli*. By maintaining a constant dilution rate while gradually increasing succinate concentration, they demonstrated enhanced tolerance to stress levels up to 160 g/L, underscoring the potential of chemostat in linking metabolic flow to mutation accumulation in steady-state cultures [17]. Additionally, the chemostat can incorporate dynamic selection pressures. The ‘morbidostat’, an experimental system developed by Toprak et al., dynamically adjusts antibiotic concentrations to continuously inhibit the growth of evolving microbial populations, effectively simulating the gradual evolutionary trajectory of antibiotic resistance under natural conditions [18].

A chemostat maintains a constant biomass concentration by dynamically adjusting the medium flow rate, preventing growth stagnation due to substrate depletion. This setup closely mirrors continuous processes used in industrial fermentation. Its primary benefit is the ability to keep microorganisms in the logarithmic growth phase, applying continuous selective pressure. Researchers continuously monitor and adjust the optical density (OD_600_) of bacteria under chemostat conditions to optimize growth and facilitate the gradual adaptation of *E. coli* populations to formic acid and CO_2_ as the sole carbon and energy sources [19]. Furthermore, Wong et al. (2018) demonstrated the expanded applications of chemostats in laboratory-based adaptive evolution. By introducing the Evolver system, they significantly broadened the scope of turbidostat use in ALE. The Evolver integrates automated control with real-time monitoring, enabling precise adjustments to medium flow rates and stress factors, alongside the online collection of key parameters such as OD_600_ and pH [20]. This integration facilitates real-time feedback and data analysis, allowing for refined management of evolutionary processes under complex stress conditions and providing robust support for the integration and analysis of multi-omics data to uncover genotype-phenotype relationships.

### 2.3 Integrated strategy for driver mutation validation

The primary goal of ALE is to identify key mutations that drive adaptive evolution through genotype-phenotype association analysis. However, the mutations generated during this process include both driver mutations, which directly contribute to phenotypic optimization, and passenger mutations, which accumulate randomly [21]. Therefore, when validating mutations, it is essential to adopt an integrated strategy combining genetics, transcriptomics, metabolomics, and bioinformatics tools to systematically analyze the functional contributions of these mutations and their regulatory mechanisms (Fig. 2d).

#### 2.3.1 Mutation reconstitution and parallel evolution experiments

Mutation reconstruction experiments are a core method for validating mutant functions. By introducing candidate mutations into the original parent strain, the individual or combined effects of these mutations on phenotype can be quantitatively assessed. In a study of glucose-limited evolution of *E. coli* MG1655, researchers introduced key candidate mutations, such as point mutations in the *rpoB* gene and an 82 bp deletion in the *pyrE-rph* intergenic region, using techniques like gene gorging. These mutations were evaluated both individually and in combination to assess their contribution to the adaptive phenotype [22]. Nine parallel evolution experiments (Parallel ALE) were conducted to identify high-frequency mutation sites through replicated evolutionary pathways, enhancing the reliability of the findings. Tenaillon et al. conducted research involving 115 *E. coli* populations over 2000 generations at a high temperature of 42.2 °C. Whole-genome resequencing revealed 1331 mutations [23], with significant convergence at the gene, operon, and functional complex levels, despite differences in mutation sites across replicated populations. This indicated that under the same selective pressure, diverse evolutionary paths could result in similar physiological adaptations.

#### 2.3.2 Integrated multi-omics analysis for deciphering mutation-phenotype associations

Whole-genome sequencing (WGS) serves as the foundation for mutation identification, but functional validation requires integrating transcriptomic and metabolomic data. In a study aimed at enhancing *E. coli* tolerance to isobutanol, the authors combined ALE experiments with transcriptomic analysis to explore the association between mutations and phenotypes through multi-omics integration. Transcriptomic analysis identified genes associated with isobutanol tolerance, and their roles were validated through gene overexpression experiments. The study demonstrated that overexpressing specific genes (e.g., *fadB*, *dppC*, *acs*) significantly enhanced *E. Coli* tolerance and production capacity for isobutanol [6]. Similarly, Cheng et al. integrated metabolomics, proteomics, lipidomics, and transcriptomics data to reveal how mutations in *E. coli* evolving with glycerol as the sole carbon source led to the reorganization of central carbon metabolism, alterations in the cofactor (NADH/NADPH) ratio, and changes in lipid composition [24]. Through this multi-omics integration, the study identified key genes and provided insights into how they adjust metabolic pathways to enhance isobutanol tolerance.

## 3 ALE as a multifaceted toolbox for *E. coli* fitness and synthetic biology innovation

**Fig. 3:**
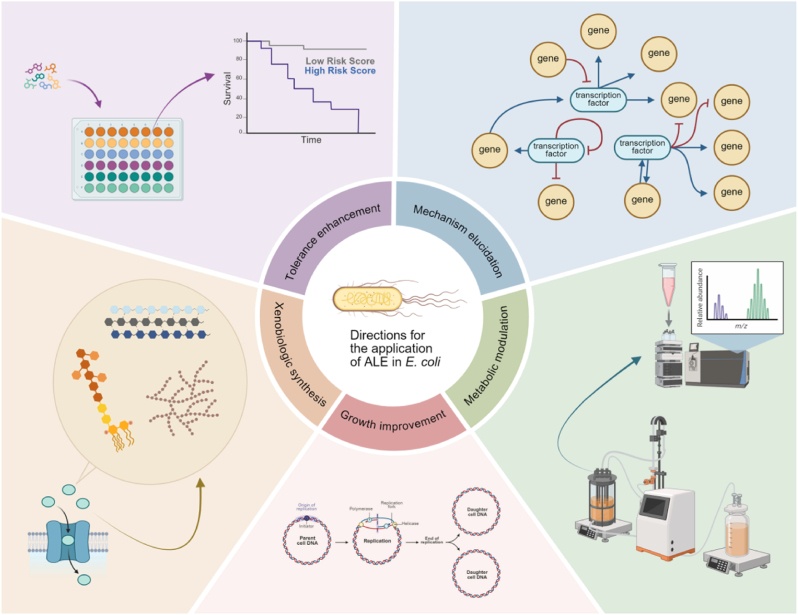
**Different application directions of ALE.** Growth improvement—optimizing genetic and metabolic pathways to accelerate proliferation; Metabolic modulation—redirecting metabolic fluxes to boost product yields and enable efficient utilization of alternative or non-native substrates; Tolerance enhancement—evolving strains to resist antibiotics and extreme environments; Mechanism elucidation—uncovering genetic and regulatory bases of adaptive responses; Xenobiologic synthesis—applying ALE to optimize incorporation of non-canonical biological components.

Researchers have employed ALE in *E. coli* across diverse conditions to optimize fitness and innovate methodologies, generally classifiable into five key application areas: growth rate enhancement, cell factory optimization, stress tolerance improvement, physiological network modulation, and xenobiological platform construction (Fig. 3).

### 3.1 ALE as a systems-level strategy for *E. coli* growth optimization

Growth rate serves as a fundamental physiological benchmark, reflecting substrate uptake efficiency, metabolic network integrity, and biomass conversion capacity. Conventional rational engineering strategies—such as genome reduction, transporter deletions, and pathway inactivation—often disrupt cellular homeostasis and reduce growth fitness. In contrast, ALE systematically coordinates multiple regulatory layers to enhance growth performance (Fig. 3, Table 1). This advantage is particularly evident where ALE compensates for engineered metabolic and genetic deficiencies: TCA cycle-impaired strains restored aerobic growth through global metabolic rewiring [25]; genome-reduced strains recovered wild-type growth rates *via rpoD*-mediated transcriptional remodelling [26]; and terminator-recoded strains overcame synthetic growth defects through adaptive optimization of translational machinery [5]. These outcomes highlight the unique capacity of ALE to uncover non-intuitive yet biologically coherent solutions that frequently evade rational design.

**Table 1:** ALE studies for growth optimization.

Strain	Condition	Span	Outcomes	Reference
MG1655 (5 repicate populations)	Glycerol-based growth medium	44 days (∼660 generations)	Mutations in genes encoding glycerol kinase and RNA polymerase lead to increased growth rates	[3]
C321.ΔA (321 stop codons recoded)	M9 minimal medium	more than 1000 generations	The growth rate is significantly higher than the ancestors	[5]
MG1655	M9 minimal medium	39∼81 days	The growth rate of the evolved strain was 1.42–1.59 times higher than that of the wild type	[22]
MS56	Flasks with M9 minimal medium	807 generations	Recovery of growth equivalent to the wild type	[26]
PB11 (PTS- mutant strain)	M9 minimal medium	120 h	The growth rate was about 330 % higher than PB11	[34]
PB11 (PTS - mutant)	In sequential batch-chemostat culture in 1-L fermenters with M9 minimal medium	80h	Specific growth rate increased by 260 % (0.42 h^−1^)	[35]
*E. coli* strains lacking the Phosphotransferase System (PTS-)	M9 minimal medium with 4 g/L of glucose	About 40 days	Rapid growth and high aromatic amino acid precursor phenotype	[37]
MG1655 (Δ*pgi* mutant)	M9 minimal medium, minibioreactors	50 days	Growth rate recovered to 46–71 % to that of wild type	[39]
*E. coli* mutants lacking three terminal oxidases	M9 minimal medium	60 days	A growth rate comparable to anaerobic wild-type, fermented glucose into d-lactic acid under aerobic conditions	[36]
dTCA (BW25113 Δ*aceA*Δ*sucA*Δ*gadA*Δ*gadB*Δ*poxB*::*acs*)	M9 minimal medium	48 days (∼230 generations)	The growth rate of the evolved strain was similar to the control strain with complete TCA cycle	[25]
MG1655	Either lactate or glycerol minimal media	60 days (l-lactate), 44 days (glycerol)	The average growth rate increased: 135 % lactic acid; 145 % glycerol	[27]
REL606	In minimal medium supplemented with 25 mg/L glucose	6000 days	The adaptation speed gradually slowed down with time	[28]
REL606 (12 repicate populations)	Glucose-limited minimal medium	20,000 generations	Overall fitness increased by 67 %; same direction of expression changes	[29]
*E. coli* B (12 repicate populations)	Glucose-limited minimal medium	20,000 generations	Rapid evolution in the first 5000 generations, and deceleration in the next 15,000 generations	[30]
*E. coli* B (12 repicate populations)	Glucose-limited minimal medium	2000 generations	The fitness of all populations increased	[31]
MG1655	Glycerol minimal medium	25 days	The growth rate increased by 60 %	[32]
MG1655	Lactate minimal medium	60 days (∼1100 generations)	The growth rate increased by 90 %	[33]

The effectiveness of ALE in growth optimization is further highlighted by convergent evolutionary patterns observed across independent experiments [14,[27], [28], [29], [30], [31]]. Parallel evolution studies consistently identify mutations in central regulatory nodes, particularly in RNA polymerase components (*rpo* genes) [22,32,33] and ribosomal proteins (*rpp* genes) [34,35], which collectively enhance transcriptional and translational efficiency while reducing energy-intensive processes such as motility [27]. This regulatory rewiring leads to coordinated metabolic adaptations, including optimized carbon uptake [34,35], redox balance adjustment [36], and energy charge maintenance [37]. The phenomenon of diminishing fitness returns—where early large-effect mutations constrain subsequent adaptive potential through epistatic interactions—provides important insights for evolutionary theory [38]. These findings collectively demonstrate that ALE not only achieves superior fitness enhancement compared to rational engineering (typically restoring or surpassing wild-type levels across various conditions) but also preserves greater genetic plasticity and metabolic balance [36,39]. This makes ALE especially valuable for constructing robust microbial platforms and exploring evolutionary dynamics.

The integration of multi-omics analyses with ALE has been crucial in deciphering these complex adaptation mechanisms. By correlating mutational profiles with phenotypic outcomes, researchers have identified key evolutionary constraints and trade-offs, enriching both fundamental microbiology and applied biotechnology. For example, mutations in components of the phosphotransferase system [28] commonly emerge in carbon-limited evolutions, illustrating how ALE naturally optimizes substrate utilization efficiency. These insights contribute to bridging the gap between laboratory evolution and precision engineering, establishing an iterative Design-Build-Test-Learn framework for next-generation strain development. This synergistic approach is particularly valuable for overcoming current challenges in complex trait engineering, where single-gene modifications often fall short of achieving the desired phenotypic outcomes.

### 3.2 ALE powered cell factory: unlocks *E. coli* metabolic agility

Microbial cell factories offer substantial advantages over traditional chemical synthesis, including milder reaction conditions, cost efficiency, and environmental sustainability. However, their industrial application is often limited by intrinsic metabolic inefficiencies and external fermentation stresses, such as the accumulation of cytotoxic substrates or products. ALE has emerged as a powerful strategy to overcome these challenges, optimizing *E. coli* chassis for improved production capacity, substrate utilization, and robustness. Through directed evolution under simulated industrial conditions, ALE not only enhances specific phenotypes but also provides fundamental insights into metabolic adaptation (Fig. 3), forming a knowledge base that informs rational engineering strategies (Table 2).

**Table 2:** ALE studies for production capacity enhancement.

Strain	Condition	Span	Outcomes	Reference
MDS42	M9 added with isopropanol (0 mM–500 mM)	24 days (∼210 generations)	Improved isopropanol tolerance	[2]
BL21 and MG1655	LB supplemented with isobutanol (starting at 0.75 % v/v, increasing to 1.2 % v/v)	144 (BL21) and 148 (MG1655) transfers	Increased tolerance to isobutanol	[6]
W3110 equipped with recombinant ALAS production	M9GTY plates with 1 g/L glycine	until biomass and 5-ALA production was stable	129 % and 205 % increase in biomass and 5-ALA respectively	[43]
ARTP-treated populations	M9P medium	60 days	Enhancement in l-cysteine tolerance; 34 % production gain	[44]
AST-4	ARTP followed by ALE,LB with gradually increased concentrations of NaAc (up to 5 g/L), NaCl (up to 0.3 M), H_2_O_2_ (up to 2 mM), and decreased pH (down to 5.5)	Until target concentration	Improved tolerance to high acetate, high osmolarity, high ROS, and acidic conditions; 53.7 % increase in astaxanthin production	[45]
KC01 (*ldhA*, *pflB*, *ackA*, *frdBC*, *pdhR*::*pflBp6*-*aceEF*-*lpd*)	LB medium supplemented with 50 g/L xylose and ethanol (gradually increase concentration from 10 to 40 g/L)	about 350 generations	The ethanol tolerance was improved twice; The ethanol yield reached 23.5 g/L	[48]
KO11	LB medium supplemented with 20–140 g/L glucose or xylose, gradually increase the ethanol concentration (35–50 g/L)	3 months	The evolved strain ly01 was able to tolerant 50 g/L ethanol. The ethanol yield exceeded 60 g/L	[49]
BW25113 *frdC*	Glycerol medium and gradually increase the ethanol concentration	78 generations	Hydrogen production increased 20 fold (0.68 ± 0.16 mmol/h), ethanol production increased 5-fold	[46]
*E. coli* W	M9 supplemented with 0.2 % (v/v) glycerol	1300 generations	Increased glycerol consumption rate and GABA production in LB with 2 % glycerol (0.39 ± 0.03 g/L, 0.08 ± 0.01 g/L/h)	[47]
*E*. *coli* W	Endpoint fermentation broth (EFB) with lysine concentrations ranging from 75 g/L to 150 g/L,GREACE assisted	7 transfers (∼1300 generations)	Improved tolerance to high lysine concentrations; Increased lysine production: 155.0 g/L	[59]
S028,TS,TSAA(GalP/Glk-dependent)	M9 minimal medium, with CRISPR/Cas9-facilitated *in vivo* mutagenesis	550 h	Improved Trp yield	[60]
*E. coli* 4HPAA-2	LB with gradually increasing 4HPAA concentration (up to 35 g/L), ARTP mutation assisted	Not explicitly stated	Higher 4HPAA production and tolerance (25.42 g/L)	[62]
BW25113	M9 minimal medium and increasing n-butanol concentrations (0.5 %–1.3 % v/v), Chemostat bioreactors	∼144 generations	Increased n-butanol tolerance and production	[61]
MG1655	M9 minimal medium supplemented with increasing concentrations of l-histidine, l-phenylalanine, l-methionine, or l-threonine	Until growth rate stabilized	Tolerance to elevated concentrations of targeted amino acids (4–6 fold increase)	[63]
PHE03 (L-Phe biosynthetic pathway reconstituted)	An initial L-Phe concentration of 20 g/L and a final concentration of 30 g/L through continuous passages	60 serial passages	Tolerance to 30 g/L L-Phe; reduced cell death rate of 36.2 % after 48 h of fermentation; The titer of L-Phe reached 80.48 g/L	[64]
W3110	Medium with increasing succinate concentration	268 days	A growth rate of 0.20 h^−1^ in the medium containing 0.592 M succinate, salt tolerance and pH shock resistance	[65]
B0016–090BB	M9 medium with glycerol as the sole carbon source; Continuous ALE (CALE) and marginal effect-assisted ALE (MEALE) under anaerobic conditions	CALE: ∼1.5 months per round, ∼800 generations over 1.5 years; MEALE: ∼6 months	Improved growth; accumulation of pyruvate; 1.07 g/L β-alanine produced	[68]
JCL260 (*E. coli* strain previously engineered for isobutanol production)	Serial transfer method in M9P medium supplemented with increasing concentrations of isobutyl acetate (IBA)	37 rounds	Increased tolerance to IBA (up to 4 g/L) and higher IBA production titers (up to 3.4 g/L)	[41]
Isobutanol producing strain	LB broth containing isobutanol	45 transfers	Tolerance of 8 g/L isobutanol	[42]
MG1655	M9 glucose medium containing 11 industrial chemicals at concentrations 60 %–400 % higher than the initial toxicity level using an automated platform	∼40 days	Increased tolerance to chemicals by 60 %–400 %; Improved production of isobutyrate and 2,3-butanediol	[40]
*E. coli* BW25113 (Parent strain: Δ*adhE* Δ*pykAF* Δ*gldA*:*:kan* Δ*pflB*::*tet)*	M9 minimal medium supplemented with 10 g/L glycerol	100.1 ± 0.3 generations	Succinate yield increased by more than 3.1-fold	[67]

A key strength of ALE is its ability to enhance both product titers and cellular robustness [[40], [41], [42]]. For instance, when applied to improve glycine tolerance in *E. coli* W3110—a critical factor for 5-aminolevulinic acid (5-ALA) production—ALE combined with pathway engineering resulted in a 205 % increase in yield [43]. Likewise, the evolution of l-cysteine tolerance from 0.2 to 20 g/L led to a 34 % production increase in fed-batch fermentation [44]. Beyond specific metabolites, ALE has proven valuable in developing strains resistant to high-density fermentation stresses, such as elevated acetate, NaCl, and oxidative stress, with one study reporting an 18.5 % increase in biomass and a 53.7 % boost in astaxanthin production [45]. Notably, ALE has also expanded the range of non-native products achievable in *E. coli* [46,47]. For instance, the evolution of the KO11 strain for ethanol tolerance [48] and production resulted in the production of over 60 g/L in 72 h—outperforming even native ethanol producers like *Saccharomyces cerevisiae* [49].

In parallel with advances in production, ALE has been equally effective in broadening substrate utilization capabilities (Table 3). For instance, evolving *E. coli* on methanol and threonine enabled autonomous growth while maintaining efficient methanol assimilation for amino acid biosynthesis [50]. Similarly, adaptation to acetate—a common inhibitory byproduct—not only improved tolerance to 40 g/L but also enhanced its use as a sole carbon source. Mutations in the *acs* gene boosted both acetate assimilation and ATP production [51,52]. These adaptations are particularly valuable for sustainable bioproduction, as seen in strains evolved for formate utilization, which holds promise for CO_2_ valorization [53], and those optimized for lignocellulosic sugars like sucrose [54] and xylose [55], where ALE overcame catabolite repression to enable co-utilization with glucose.

**Table 3:** ALE studies for substrate utilization improvement.

Strain	Condition	Span	Outcomes	Reference
Methylotrophic *E. coli*	M9 minimal medium with 250 mM methanol	18 passages	Improved methanol utilization	[50]
*E*. *coli* introduced formate assimilation pathway	Modified EMK medium	60∼150 serial subcultures	Improvement of formate utilization, ethanol production in 90 mg/L formate sugar free system	[53]
*E. coli* lacking four genes involved in acetylCoA consumption (*adhE*, *pta*, *ldhA*, *andfrdA*)	M9 containing 5g/L acetate	40 days	Improved acetate utilization efficiency and growth rate	[51]
*E. coli* derived from MG1655: Δ*pflB*, Δ*adhE*, Δ*frdA*, Δ*xylFGH*, evolved	Mineral medium with xylose as the sole carbon source (40 g/L to 120 g/L)	15 transfers	Improved tolerance to acetate and productivity of ethanol or lactate	[52]
MG1655 introducing the sucrose utilization pathway gene *csc* and *E. coli* W	M9 minimal media with 20 g/L sucrose	40 days	Improved sucrose utilization	[54]
W3110 Δ*ptsH*, *ptsI*, *crr::kmR*	M9 medium supplemented with 0.2 % glucose, anaerobic conditions	Not explicitly stated	The ability to co-utilize glucose-xylose; improved ethanol production; restored anaerobic growth	[55]
MLB (MG1655 Δ*pflB* Δ*ldhA*)	M9 medium with 10 g/L NaAc as the sole carbon source	Until the growth rate became stable	Increased succinate production and tolerance during anaerobic phase (111 g/L, 0.74 g/g glucose)	[56]
MG1655	M9 supplemented with different carbon sources: first evolved from l-lactate to glycerol, and vice versa	1400∼1600 generations	General growth advantage and higher acetyl-CoA productivity	[57]
MG1655-derived strain with a hybrid methanol assimilation pathway	M9 minimal medium with 400 mM methanol, 1 g/L yeast extract	28 transfers	Improved methanol assimilation and utilization	[66]
MG1655-derived strain with *xylFGH* gene deleted	Mineral AM1 medium supplemented with xylose as the sole carbon sourc (40∼120 g/L)	15 transfers	Xylose consumption rate doubled, lactate productivity increased by 50 %	[58]

ALE often induces comprehensive metabolic rewiring that enhances the concurrent utilization of mixed substrates [54,55]. For instance, aerobic evolution on acetate improved succinate production during anaerobic fermentation, achieving 111 g/L through enhanced TCA cycle flux [56]. In another example, evolution on l-lactate and glycerol yielded strains with improved acetoin production, attributed to the synergistic upregulation of glycolysis and gluconeogenesis genes (*glpK*, *ppsA*) [57]. These cases highlight the ability of ALE to uncover non-intuitive solutions, such as the identification of *gatC* as a novel xylose transporter in evolved strains [58], a discovery difficult to predict through rational design alone.

The integration of ALE with high-throughput omics and synthetic biology tools has further accelerated strain optimization. Techniques like GREACE [59] and CRISPR-Cas9 [60] mutagenesis have significantly reduced evolutionary timelines while enabling precise phenotypic improvements, as exemplified by lysine-tolerant mutants enriched within only seven serial transfers and tryptophan-producing strains with 19.7 % higher yields. Biosensor-driven selection, utilizing fluorescent reporters or metabolite sensors, has streamlined the isolation of high-performing variants [61,62], while multi-omics analysis has illuminated the genetic basis of adaptations—from membrane transporter modifications to global regulator mutations [2,[63], [64], [65]].

The functional significance of previously unexplained mutations emphasizes the importance of comprehensive genetic characterization. By combining empirical evolution with targeted design [[66], [67], [68]], ALE not only resolves immediate production bottlenecks but also serves as a discovery platform for fundamental biological insights, solidifying *E. coli* as a versatile chassis for next-generation biomanufacturing.

### 3.3 ALE-driven insights into *E. coli* antibiotic resistance and environmental adaptation

ALE has emerged as a powerful methodology for systematically investigating the adaptive responses of *E. coli* to antimicrobial and environmental stresses (Fig. 3), offering critical insights into evolutionary mechanisms with both clinical and biotechnological significance (Table 4, Table 5). By applying controlled selection pressures ranging from antibiotic exposure to extreme physicochemical conditions, ALE, combined with multi-omics analyses, has revealed both conserved and stress-specific adaptation strategies, shedding light on fundamental microbial evolutionary principles.

**Table 4:** ALE for drug resistance.

Strains	Conditions	Span	Outcomes	Reference
MG1655 and Δ*recA* mutant	LB containing 10 g/L NaCl with increasing concentrations of ciprofloxacin and enrofloxacin starting from one fourth of the minimum inhibitory concentration (MIC)	30 transfers	MG1655: Ciprofloxacin 256 μg/mL, enrofloxacin 512 μg/mL. Δ*recA*: Ciprofloxacin 4 μg/mL, enrofloxacin 32 μg/mL	[70]
*E. coli* 307	LB and M9 medium with two antibiotic concentration gradients: spatial gradient-microfluidic gradient chamber and temporal gradient-homogeneous batch continuous culture	over 5 days	Higher resistance compared to MGC	[71]
MG1655 pre adapted in M9G medium	Two adaptation pressures to the MIC of benzalkonium chloride in M9G: survival - removed after 4 h of exposure, survival - removed after 22 h of exposure	∼150 generations	Both treatments showed reduced lag times and weak adaptation to antibiotics	[74]
MG1655 (WT), MG1655 + mutagen, *mutD* Δ*mutL*::*zeoR* (mutator strain)	M9 minimal media with ciprofloxacin or colistin, mutagenesis by nucleotide analogs or genetically	18 days	Resistance to ciprofloxacin and colistin; increased mutation rate	[72]
MG1655	Mueller-Hinton broth II (MHBII) with amikacin (AMK), aiperacillin (PIP), tetracycline (TET). Selection regimes: Gradient: 2-fold antibiotic gradient in 10 dilutions; Increment: 100 %/50 %/25 % daily increase in drug concentration	14 days	AMK: up to 512 mg/L; PIP: up to 192 mg/L; TET: up to 15 mg/L Increased growth rate, collateral sensitivity and cross-resistance	[73]
Mds42 (pre adapted to modified M9)	High throughput evolution was performed in M9 supplemented with 95 antimicrobial chemicals	Reaching half maximal inhibitory concentration	Drug resistance, cross resistance, and collateral sensitivity	[69]
MG1655 with a CREATE-based global regulator library	M9 with furfural (0.9 g/L to 3.2 g/L)	27 days (53 transfers)	Evolved strain tolerates up to 4.7 g/L furfural, also shows significant cross-tolerance	[81]

**Table 5:** ALE for tolerance to physical perturbation.

Strain	Conditions	Span	Outcomes	Reference
MG1655	M9 glucose minimal media at 42 °C	20 days	Relative fitness increased at 42 °C	[12]
MG1655 *zba::kan*	LB media, gradually increase the culture temperature	620 generations (∼2 years)	A maximum growth temperature of 48.5 °C	[75]
REL606 (unable to use arabinose)	Davis minimal medium with 25 g/mL glucose at 41.5 °C	2000 generations	Following acclimation at 41.5 °C, 2/3 lines exhibited improved survvival at 50 °C	[76]
MG1655	Evolugator culture chamber containing LB. Temperature was increased from 44 °C to 49.7 °C	2 months	A thermophilic descendant from MG1655	[77]
606P and 607P (REL606 and REL607 pre-evolved at 37 °C for a month)	M9 minimal media, under different fluctuating temperature regimes between 15 and 43 °C (slow/fast periodic, random), BioSan LV Personal Bioreactor RTS1-C	∼600 generations	The evolution of specialists was favored in the random regime, while generalists were favored in the periodic regimes; Phenotypic restoration at 37 °C; Increased growth rates at both 15 °C and 43 °C for generalists	[79]
MG1655	LB supplemented with glucose (11 mM) and HEPES buffer (100 mM, pH 7.5), pressure gradually increased from 41 MPa to 62 MPa.	505 generations	The ability to grow at 62 MPa; Extended lag phase at 60 MPa (20 h)	[80]
MG1655 (pre-evolved on glucose minimal medium)	M9 glucose minimal medium (0.2 mM MgSO_4_, 150 mM MES buffer). pH: 5.5. Automated system	800 generations	Increased fitness at pH 5.5 (growth rate of 0.83 h^−1^ compared to 0.67)	[78]

Comparative analysis of antibiotic resistance evolution reveals that *E. coli* utilizes both general and compound-specific adaptation mechanisms. Mutations in transport systems and porins are common resistance strategies across various antimicrobial classes (e.g., β-lactams, tetracyclines) [69], while the SOS response is particularly critical in fluoroquinolone resistance, with even subtle antibiotic structural variations significantly influencing evolutionary trajectories [70]. These adaptations often involve fitness trade-offs—enhanced resistance frequently correlates with collateral sensitivity to secondary agents [69]. Advancements in methodologies, such as microfluidic gradient systems [71] and Evolutionary Action analysis [72], have refined our ability to distinguish driver mutations from neutral variants. Studies examining *E. coli* responses to gradual versus stepwise increases in antibiotic stresses reveal that gradual stress escalation results in more reproducible adaptations than abrupt challenges [73]. Schmidt et al. further elucidated the stress mechanisms of strains under abrupt and constant antibiotic stress [74].

At the environmental stress level, thermal adaptation studies highlight the remarkable plasticity of *E. coli* proteostasis network. Chaperone mutations (DnaK, GroEL) frequently mediate thermal tolerance [[75], [76], [77]], although these adaptations typically reduce fitness at standard temperatures, reflecting the evolutionary constraints imposed by protein stability trade-offs. Similar compromises occur in acid adaptation, where membrane remodelling improves proton exclusion but may impair nutrient uptake efficiency [78]. Notably, parallel evolution experiments show that while initial adaptive mutations vary across genetic backgrounds, they often converge on similar functional networks (e.g., transcriptional regulation, membrane composition) [12,79,80], suggesting constrained evolutionary solutions to physicochemical challenges.

The integration of cutting-edge tools has significantly improved the resolution and throughput of ALE [12,77,81]. However, current studies are still limited by a focus on single stressors, which fail to replicate the multifactorial nature of natural environments. Future research should prioritize combinatorial stress regimens and develop predictive models that consider genetic background effects. These efforts will be crucial for translating laboratory findings into clinical antimicrobial strategies and robust industrial strains. Such advancements will require closer integration of high-throughput phenotyping with systems-level modelling to bridge the gap between single-gene effects and emergent network properties.

### 3.4 Harnessing ALE to decipher and optimize *E. coli* physiological networks

While *E. coli* is the most extensively characterized model organism in microbiology, current understanding of its dynamic physiological responses to artificial interventions remains surprisingly incomplete (Fig. 3). ALE has emerged as a powerful tool to bridge this knowledge gap, providing unique insights into the remarkable adaptive capabilities of this organism while also revealing critical limitations in current methodologies (Table 6).

**Table 6:** ALE for physiological mechanism modulation.

Strains	Conditions	Span	Outcomes	Reference
EG1 and EG2 (Δ*epd* Δ*gapA* strains, pyridoxine auxotrophs) and P3P strain (glycerate auxotroph, Δ*pgk*, Δ*patZ*)	M9 minimal medium with 5 mM glycerol, 20 mM succinate (for EG1 and EG2) or 10 mM glycerol, 10 mM succinate (for P3P)	∼50 h for EG1and EG2, 6 weeks for P3P	*E. coli* recruits NAD(P)-dependent succinate semialdehyde dehydrogenase to replace erythritose 4-phosphate dehydrogenase and glyceraldehyde 3-phosphate dehydrogenase to alleviate pyridoxine dystrophy	[82]
Five metabolic gene knockout MG1655-uPtsHlcrr (Δ*ptsH*, Δ*ptsI*, Δ*crr*), uSdhCB (Δ*sdhC*, Δ*sdhA*, Δ*sdhD*, Δ*sdhB*), uTpiA (Δ*tpiA*), uPgi (Δ*pgi*), uGnd (Δ*gnd*)	Glucose minimal media; Automated platform	∼40 days	Remodelling metabolic networks to compensate for the fitness deficit	[83]
Four types of metabolical enzyme deficient *E*. *coli* (Δ*pgi*, Δ*ppc*, Δ*pta*, Δ*tpi*)	M9 minimal medium	30∼50 days	Evolved strains improve fitness by upregulating active pathways	[84]
Four types of *E*. *coli* containing unbranched electron respiratory chains with different proton production efficiencies	M9 minimal medium	∼400 or ∼700 generations	The metabolic flux of complex II of ETS or ED pathways is adjusted to obtain an optimized growth rate	[85]
Nissle 1917	M9 with 30 g L^−1^ allulose. Automated device.	∼400 generations (ALE) + additional rounds of FADS	The ability to utilize allulose as sole carbon source	[86]
*E. coli* with deactivated methyl-mismatch repair system (Δ*mutS*)	M9 minimal medium supplemented with either 0.2 % (w/v) of lactate or 0.2 % (v/v) glycerol as the sole carbon source	∼800 generations	Faster mutation accumulation rate and general fitness advantages in heterogeneous environments	[87]
*E. coli* C600-pL53T	Antibiotic-free LB broth	∼600 generations	Deletion of plasmid anti-SOS gene psiB reduces plasmid transfer ability; the inactivation of genomic transcription suppressor SspA promotes the optimization of host fitness	[88]

At the metabolic level, ALE studies have highlighted *E. coli* extraordinary capacity for network rewiring, particularly in response to genetic perturbations. The functional promiscuity of catalytic sites and compensatory flux redistribution observed in knockout strains underscore the metabolic plasticity that has contributed to the evolutionary success of this organism [[82], [83], [84], [85]]. Additionally, the discovery of new enzyme functions through evolution holds significant promise for expanding metabolic engineering tools [82]. The successful integration of non-canonical substrates, such as d-allulose, into central metabolism further underscores the potential for biotechnological applications [86]. However, these findings raise critical questions about the stability and efficiency of such adapted states. While short-term adaptations are well-documented, the long-term maintenance of these novel metabolic configurations across generations remains poorly understood. Furthermore, some uncharacterized mutational effects underscore the need for a more systematic exploration of the associated fitness trade-offs.

Genetic studies using ALE have also yielded fundamental insights, though not without important caveats. The accelerated adaptation observed in *mutS*-deficient strains clearly demonstrates the evolutionary advantage of increased mutation rates [87], yet this advantage comes at the cost of genomic stability, which may limit practical applications. Similarly, plasmid evolution experiments have shed light on the delicate balance between resistance acquisition and fitness costs, yet these findings may not fully capture the complexity of horizontal gene transfer in natural environments [88]. Collectively, these examples highlight a significant gap in ALE research: the extent to which laboratory-evolved strains authentically replicate natural adaptation processes.

### 3.5 ALE as the crucible for xenobiotic life

Xenobiology, an emerging subfield of synthetic biology [89], seeks to reprogram the central dogma by systematically incorporating non-canonical biological components—such as unnatural amino acids (ncAAs), xenonucleic acids (XNAs) containing rare elements (e.g., fluorine, sulfur) or specialized groups (e.g., azides) [90], and artificial metabolic pathways [91]—toward the creation of truly synthetic life forms (Fig. 3). These engineered systems offer distinct advantages: residue-specific modifications allow fine-tuning of enzymatic properties like substrate binding, stability, and catalytic activity; orthogonal translation systems reduce metabolic burden while preventing interference with native processes, thus enabling the production of compounds that are difficult to synthesize and resistant to degradation [92]; and auxotrophic designs offer robust biocontainment strategies [[93], [94], [95]]. Furthermore, advancements in xenobiology have significant implications for expanding biomolecular chemical diversity and exploring the origins of life. However, challenges such as host toxicity and inefficient integration of non-canonical components remain.

ALE has proven instrumental in overcoming these limitations in *E. coli*. Through progressive adaptation under controlled selective pressures, ALE enhances host tolerance to xenobiological stress while optimizing substrate uptake and integration kinetics. For example, Trp-auxotrophic *E. coli* subjected to ALE under gradient concentrations of the Trp analogue [2,3]Tpa evolved independence from Trp and achieved genome-wide replacement of Trp with [2,3]Tpa [96]. This evolutionary strategy synergizes effectively with two primary ncAA incorporation techniques—selective pressure incorporation (SPI) and stop codon suppression (SCS) [97].

SPI leverages the endogenous translation machinery of auxotrophic hosts in media with gradually increasing ncAA concentrations to achieve proteome-wide modifications [98,99]. The most extensively studied application of SPI is Trp substitution, including the evolved utilization of fluorinated indole precursors [[100], [101], [102], [103], [104]] and Trp→ [2,3]Tpa replacement [[105], [106], [107]]. Notably, Bacher et al. demonstrated the adaptive evolution of proteomes to chemical ambiguity using a phage model, providing the first experimental evidence that minimal mutations enable complex proteomes to accommodate unnatural amino acids [104]. Marlière et al. used a patented dual-chamber system with pulsed nutrient feeding to circumvent substrate retention from bacterial wall adhesion, achieving 90 % genomic chlorodeoxyuridine substitution in *E. coli* through precisely regulated differential nutrient supply. This breakthrough offers essential technical support for reliable genomic recoding [108].

In contrast, SCS relies on engineered aminoacyl-tRNA synthetase (aaRS)/tRNA pairs to competitively suppress termination signals, enabling site-specific protein labelling, while ALE further enhances nonsense suppression efficiency [109,110]. Thyer and Ellington expanded the understanding of the SCS toolkit by elucidating the core role of tRNA as a “dual decoder” in genetic code expansion—enabling both codon-anticodon pairing and aaRS specificity [111].

Recent studies are integrating genome editing with adaptive evolution to address key industrial bottlenecks. The Heidari-Budisa platform utilized Tn7-like transposases for the genomic integration of orthogonal systems, eliminating plasmid instability while combining SPI to enhance proline analogue incorporation [112]. Simultaneously, the development of *in vivo* ncAA biosynthetic pathways is reducing dependence on expensive chemical precursors and continuous feeding [[113], [114], [115]]. Additionally, CRISPR-assisted mutagenesis coupled with microfluidic screening platforms is set to accelerate next-generation xenobiological systems, while cell-free evolution strategies may bypass membrane transport limitations [91]. Li et al. used flexizyme to achieve tRNA acylation and N-terminal incorporation of 32 noncanonical monomers, establishing the first design rules for catalytic tRNA acylation (structural similarity, electronic effects, steric constraints) to enable systematic genetic code reprogramming and non-natural biopolymer synthesis [116]. Collectively, these advancements position ALE not only as an optimization tool but also as a critical methodology bridging xenobiology theory with scale-up applications.

## 4 Integrated platforms for MAM-ALE

**Fig. 4:**
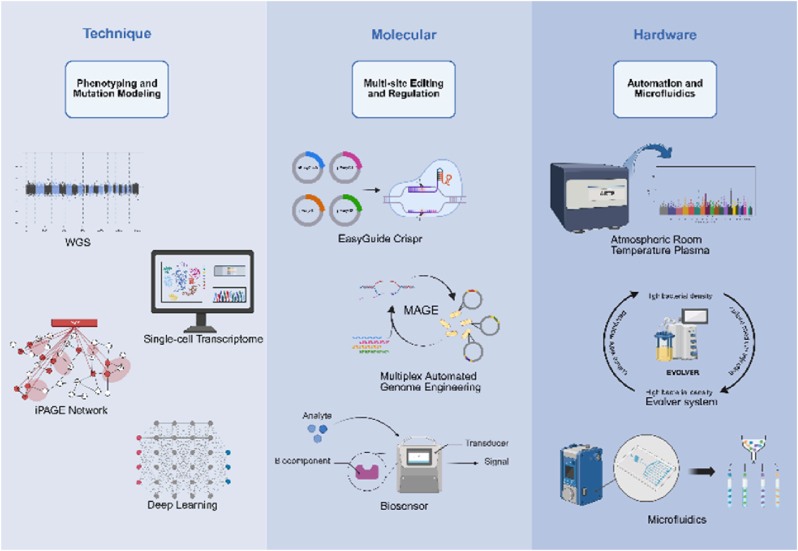
**Multi-dimensional technological convergence of MAM-ALE in prokaryotic evolution.** An overview of advanced phenotyping and computational approaches, multi-site genome editing tools, and automation/microfluidic platforms. These combined methodologies enable high-throughput, precise, and adaptive evolution strategies in *E. coli*.

In recent years, the integration of ALE with bioinformatics, molecular biology, and synthetic biology techniques has profoundly reshaped the paradigm of prokaryotic research (Fig. 4). This convergence has given rise to *MAM-ALE* (Machine learning-Automated hardware-Molecular biology enhanced ALE), a unified methodology that combines three pillars: [M]achine learning prediction, [A]utomated bioreactor control, and [M]olecular intervention tools. MAM-ALE merges predictive bioinformatics with automated hardware and CRISPR-based genome editing within closed-loop systems. Machine learning (ML) models analyze real-time omics data to guide evolutionary processes in automated bioreactors, while molecular tools enable precise interventions. This integration accelerates evolutionary studies, reducing timelines from months to weeks, and extends the ability to engineer non-growth-coupled traits. Demonstrated in industrial contexts through evolved strains with specialized metabolism, this approach offers enhanced precision for probing adaptation mechanisms and designing industrial microbes, marking a significant evolution in laboratory evolution methodologies.

### 4.1 The co-evolution of bioinformatics and omics technologies

WGS and the development of mutation hotspot databases have generated high-resolution mutation maps to facilitate ALE [3]. For example, the *E. coli* mutation hotspot database, based on WGS, has identified conserved high-frequency mutation sites (e.g., *rpoB* and *arcA*) across evolutionary stages, often linked to the regulation of core carbon metabolic flux [117]. By integrating the whole-genome computational framework of interactive Pathway Analysis and Gene Enrichment (iPAGE), researchers can simulate the dynamic effects of mutations on the global metabolic network. Goodarzi et al. (2010) employed the iPAGE framework for fitness analysis, revealing that specific mutations in *E. coli* loci (such as *adhE* and *acrAB-tol*C) under ethanol stress significantly improved strain tolerance by enhancing ethanol degradation and membrane stability [1]. This finding laid the groundwork for standardized targeted mutagenesis strategies.

The incorporation of deep learning and evolutionary prediction models enhances ALE data modelling capacity. Wang et al. (2018) integrated 83 feature variables, including strain genetic background, culture medium components, and stress conditions, to construct the first comprehensive *E. coli* whole-genome mutation map. This work illuminated the intricate connections between environmental stress and gene mutations [117]. Using this dataset, artificial neural network (ANN) and support vector machine (SVM) models were combined into a unified prediction framework, enabling successful probabilistic identification of gene-level mutation targets. This advancement addresses the limitations of traditional static metabolic models. For example, in citric acid production strain optimization, the predictive model informed the directed evolution of MVA pathway-related genes, such as HMGS and HMGR [118].

The integrated analysis of multi-omics datasets provides a comprehensive view of complex phenotypes. Cheng et al. (2014) demonstrated through a joint analysis of the transcriptome and metabolome that variations in carbon metabolic fluxes (e.g., *rpoC* and *glpK*) among *E. coli* subpopulations influenced their glycerol utilization adaptability, revealing two independent yet complementary growth optimization mechanisms [24]. In future research, single-cell RNA sequencing (scRNA-seq) and spatial proteomics technologies will offer dynamic insights at the single-cell level, shedding light on how population heterogeneity affects evolutionary outcomes during the ALE process [119].

### 4.2 The precision regulation revolution in molecular biology technology

Traditionally, the time scale of ALE experiments is measured by the number of generations or CCD. Research has shown that in long-term ALE experiments with *E. coli*, subpopulations with prolonged division times often accumulate mutations linked to upregulation of metabolic flux (e.g., *rpoB* and *pyrE*), while subpopulations with faster division times tend to retain the original phenotype. This differentiation highlights the importance of tracking division history as a key factor in evolutionary selection [16]. Current methods for tracking *E. coli* adaptive evolution mainly involve recording passage numbers or estimating division counts, which lack precision in determining the exact number of cell divisions. Advanced technologies enable the cultivation of individual *E. coli* cells on microfluidic chips and allow the labelling of cell cycle-related proteins (e.g., FtsZ protein) with GFP tags, facilitating the quantification of division times and monitoring of evolutionary processes. Notably, Sebastian Jessberger's team at the University of Zurich developed the inducible cell division counter (iCOUNT), which tracks the division history of individual cells in complex tissues of mice and humans by monitoring cyclin H3.1 expression changes through endogenous markers [120]. This principle may be adapted for monitoring cell division history in *E. coli* ALE in the future.

The integration of high-throughput gene editing technologies, such as MAGE, with ALE has greatly accelerated the evolutionary process. In the adaptive study of artificially recoded *E. coli* C321, MAGE was used to reconstruct alleles like *prfB* and *prfC* in ancestral strains. Combined with ALE, this approach enhanced the strain's growth robustness in complex media and provided in-depth physiological insights into reprogrammed *E. coli* [5]. Recently, the CRISPR-EasyGuide system developed by Gross et al. (2025) has simplified the screening of multiple gene mutations. In sucrose-utilizing strain optimization, this system identified regulatory effects of cooperative mutations in the *glf* and *glk* genes on carbon metabolic flux *via* reverse metabolic engineering [121]. These technologies can also be integrated with metabolite-responsive biosensors to achieve real-time coupling of environmental stress and gene expression [122]. For example, in developing 4-hydroxy-phenylacetic acid (4-HPAA)-tolerant strains, combining a quorum sensing regulatory module with an mCherry reporting system has significantly improved the dynamic screening efficiency of products [62].

### 4.3 Closed-loop design strategy of synthetic biology and ALE

The integration of synthetic biology and ALE is enabling a seamless bridge between rational design and evolutionary optimization. Using the genomic streamlined strain MDS42 as an example, ALE has addressed metabolic deficiencies overlooked in rational design, such as the absence of the acetate assimilation pathway. This highlights the importance of bypass metabolism in the design of minimal genomes [26]. Additionally, the fusion of gene circuit rational design with ALE allows for the reverse engineering of evolutionary pathways. For example, by combining adaptive evolution with transcriptomics, researchers have clarified the genetic underpinnings of isobutanol tolerance in *E. coli*, advancing the development of robust strains through transcriptomics-based reverse engineering [6]. In the optimization of non-natural metabolic pathways, similar strategies have led to significant advancements in developing autotrophic *E. coli*. By knocking out key heterotrophic enzymes like phosphoglucose isomerase (*pgi*) and glucose-6-phosphate dehydrogenase (*zwf*) and introducing CO_2_ fixation pathways, such as the CBB cycle, alongside ALE screening, an Israeli research team successfully converted *E. coli* into an autotrophic organism, relying solely on CO_2_ for growth [7]. This accomplishment pushes beyond traditional metabolic engineering boundaries and sets a new paradigm for sustainable biomanufacturing.

In industrial applications, the collaborative innovation of physical mutagenesis and ALE has shown considerable advantages through technical integration. For instance, combining heavy ion radiation mutagenesis with ALE has significantly enhanced *E. coli* tolerance to l-cysteine. Omics analysis reveals that radiation-induced genomic instability, combined with ALE selection pressure, synergistically drives the upregulation of the *eamB* gene, resulting in a 2.2-fold increase in product titer [44]. These methodologies not only validate the effectiveness of integrated technology but also provide a robust framework for strain optimization in complex industrial contexts.

### 4.4 Trends in the integration of ALE with emerging technologies and multidisciplinary intersections

With the maturation of ALE technology and the deepening of interdisciplinary integration, three emerging trends are reshaping its methodological framework and expanding its application: ML-driven closed-loop experimental design, real-time evolutionary tracking at single-cell resolution, and synergistic evolutionary regulation at the microbial community level. These trends overcome traditional ALE limitations in spatiotemporal scales and system complexity, offering an unprecedented engineering platform for synthetic biology and biomanufacturing (Fig. 4).

In bioinformatics-assisted ALE design, the integration of whole-genome mapping with deep learning models has overcome the static limitations of traditional metabolic models. LaBella et al. unveiled new insights into yeast evolution by analyzing a dataset of over 900 yeast genome sequences using artificial intelligence, paving the way for future ML-driven dynamic evolutionary design [123]. Recent research further highlights that the ProEnsemble machine learning framework enables the automated, synchronous evolution of multiple key genes in metabolic pathways, combining automation technology with machine learning. This approach addresses the challenge of replicating thousands of years of natural evolution in a much shorter timeframe and with fewer iterations [124].

The resolution of ALE tracking has been enhanced through the integration of single-cell omics and high-throughput instruments. The microfluidic device based on the Microbe-seq system, coupled with computational-assisted whole-genome sequencing, enables the analysis of population heterogeneity and single-cell characteristics. This technology is expected to play a significant role in adaptive evolution research in the future [125]. Additionally, adaptive evolution combined with flow cytometry-assisted high-throughput screening has led to the successful selection of non-genetically modified yeast strains with elevated protein content, achieving the highest reported protein levels to date [126].

The integration of synthetic biology with ALE is expanding from single strains to microbial communities. Zhang et al. explored the adaptive evolution strategies of probiotics under selective pressures from different host intestines by combining shotgun metagenomic sequencing and whole-genome resequencing. Their work elucidated the co-evolution mechanism of intestinal microbiota following probiotic introduction, offering a novel approach for screening mutant strains tailored for the intestine [127]. This strategy holds particular value in complex scenarios, such as environmental stress resistance evolution in microbiota; for example, ALE can improve the acid resistance of microbial communities, expanding the applicability of thermoacidophiles [128].

Looking ahead, the integration of spatial omics with automated fermentation platforms is set to enhance the precision of ALE. scRNA-seq can uncover the spatiotemporal distribution of subgroup-specific mutations, such as soil microbial drug resistance regulated by TCA cycle flux [129], within population dynamics. Simultaneously, robotically controlled continuous culture systems can conduct hundreds of parallel evolution experiments, providing ultra-high-throughput datasets for machine learning models [130]. The cross-integration of these technologies marks a new phase in ALE characterized by “design-evolve-validate” integration, with promising applications in synthetic lifeform construction and sustainable biomanufacturing.

## 5 Conclusion and outlook

The ALE workflow—“principles and design → mutation-driven evolution → screening → functional validation”—has undergone substantial refinement, giving rise to several transformative trends. Recent advancements show that multifactorial evolution systems and microbial consortia more accurately replicate organism-specific adaptive patterns within defined environmental and genetic contexts. The integration of ML algorithms is shifting the focus from phenomenological observation to predictive modelling, significantly reducing experimental redundancy and enhancing the precision of evolutionary forecasting.

Technological innovations are revolutionizing ALE implementation. Automated hardware platforms, coupled with advanced biomutagenesis tools, have markedly accelerated adaptive evolution timelines. Meanwhile, multi-omics, high-throughput screening, and single-cell technologies enable unprecedented resolution in tracking divergent evolutionary trajectories across parallel experiments. These advances, combined with breakthroughs in molecular biotechnology, support comprehensive functional validation of multi-site mutations, facilitating the transition of ALE from system-level co-evolution to protein-scale precision engineering. Notably, the synergistic integration of ALE with rational design strategies has emerged as a powerful approach for phenotype optimization, improving efficiency while reducing costs.

As a cornerstone methodology in the Design-Build-Test-Learn cycle of synthetic biology, ALE bridges the gap between directed evolution and rational design paradigms. Its continued advancement promises more reliable translation of evolutionary findings into both theoretical and practical applications. Looking forward, ALE is set to drive ongoing methodological and technological advancements, establishing it as an indispensable tool for next-generation biotechnological innovation.

## CRediT authorship contribution statement

**Weixiang Peng:** Writing – review & editing, Writing – original draft, Validation, Data curation, Conceptualization. **Xi Zhang:** Writing – review & editing, Validation, Data curation, Conceptualization. **Qingsheng Qi:** Writing – review & editing. **Quanfeng Liang:** Supervision, Project administration, Funding acquisition, Conceptualization.

## Declaration of competing interest

The authors declare that they have no known competing financial interests or personal relationships that could have appeared to influence the work reported in this paper.

## Data Availability

Data will be made available on request.
